# Can the improvement of the social credit environment enhance corporate ESG scores?

**DOI:** 10.1371/journal.pone.0300247

**Published:** 2024-03-26

**Authors:** Chao Han, Baoqi Chen

**Affiliations:** School of Economics, Shandong University of Fiance and Economics, Jinnan, China; University of Malta, MALTA

## Abstract

The ESG scores of corporations is a crucial manifestation of their long-term strategic goals, attracting significant attention from society. The impact and underlying mechanisms of the enhancement of the social credit atmosphere on the ESG performance of corporations remain unclear. This study utilizes a sample of Chinese A-share listed companies from 2010 to 2020, employing the Difference-in-Differences (DID) methodology to investigate the relationship of the establishment of the social credit system on company ESG scores. This study reveals that the establishment of the social credit system significantly advances corporate ESG scores. Heterogeneity results indicate that the positive effect is more pronounced in state-owned enterprises or companies having substantial institutional shareholding. Furthermore, the implementation of the social credit system amplifies corporate ESG scores through three key mechanisms: fostering green technology innovation, cultivating ethical and moral corporate cultures, and optimizing the overall business environment. This paper enriches the informal institutional researches about the driving factors of corporate ESG scores, providing valuable insights for policymakers and corporate decision-makers.

## 1 Introduction

ESG emerged as an extension of Corporate Social Responsibility (CSR) [1], and was first proposed in the United Nations Global Compact in 2004. The primary aim is to foster global sustainable development. Although ESG and CSR evaluations share similarities, they differ in their focus. While CSR mainly focuses on corporate social responsibility (S), ESG focuses more on the strategic layout and sustainable development of the enterprise, and the contents are broader, which reflects how enterprises and market investors integrate environmental (E), social (S), and governance (G) with the enterprise’s business activities [2]. ESG, as an important measure of corporate non-financial performance, has gradually become the focus of society as well as enterprises in the context of China’s economic transformation and high-quality development [3]. In particular, ESG provides systematic and comprehensive information regarding both environmental and social aspects, enabling more informed investment decisions and promoting high-quality enterprise development [4]. One of the issues that ESG focuses on is the environment, which has now received the attention of most studies [5, 6]. Furthermore, prior research has demonstrated that a well-established ESG framework can enhance firms’ reputation and image in the market [7], further release positive signals, alleviate information asymmetry [8], promote stock liquidity [8, 9], and enhance firms’ financial performance [10], thus enhancing the competitive advantage of enterprises in the market and promoting their long-term development. The economic effect of corporate ESG performance enhancement is not limited to financial performance, but also has an impact on corporate human capital. The good performance of corporate ESG is beneficial for minimizing human capital wastage and addressing labor shortage concerns [11]. Therefore, it is of great practical significance to examine the factors that drive corporate ESG performance, which can help to provide guidance for corporate ESG performance enhancement as well as social sustainable development.

Current research predominantly examines the driving factors behind corporate ESG scores from a micro-level perspective, such as board diversity characteristics [12], board gender structure [13], CEO’s hometown background [14], firm size [15], and financial status [16]. Research focusing on the external environment of corporations is relatively scarce and often confined to formal institutional frameworks. For instance, existing studies have examined the impact of external institutional factors including tax incentives [17, 18], green technological innovation [19], green credit policies [20], and capital market openness [21] on corporate ESG scores. However, there is currently a literature gap in studying the driving factors of corporate ESG scores from the perspective of informal institutions. Social credit, as an informal system, includes many factors such as market, culture, and rule of law, and is an important aspect of the corporate external environment, which largely influences the decision-making behavior and development level of corporations [22]. A favorable credit environment contributes to the establishment of social moral norms, which, in turn, constrains corporate behavior, reducing unethical behaviors such as corporate violations [23] and tax avoidance [24]. The Agency theory suggests that the corporate ESG scores is characterized by a long-term cycle and high investment, which may pose a contradiction to the maximization of shareholder interests. Moreover, Cui et al. (2023) [25] suggest that the pilots of the social credit system contribute to strengthening the constraints on managerial behavior, alleviating the principal-agent conflict, and promoting the disclosure of environmental information by corporations. Therefore, a high-quality social credit level helps corporations fulfill their environmental protection responsibilities, reduce corporate violations such as tax avoidance, adopt more ethical corporate behaviors, and pay more attention to environmental, social, and governance performances to gain a long-term competitive advantage. However, existing relevant studies have not found the impact of the credit environment on corporate ESG scores and the underlying mechanisms.

Because of this, based on the quasi-natural experiment of "social credit system construction", this paper selects Chinese A-share listed companies from 2010 to 2020 as the research sample, to empirically examine the impact of the improvement in the social credit environment on corporate ESG scores, and to try to explain the underlying mechanism. Firstly, we utilize the Difference-in-Differences (DID) method to conduct the foundational examination of the impact of social credit system construction on corporate ESG scores. We also perform a series of robustness tests to ensure the reliability of the baseline results. Secondly, we focus on three channels, namely green technological innovation, ethical and moral culture, and the optimization of the business environment, to examine the mechanisms through which the credit environment influences ESG performance. Furthermore, taking into account differences in corporate ownership and institutional shareholding, we further analyze the heterogeneity of the impact of credit improvement on corporate ESG scores.

The possible marginal contributions of this paper compared to the existing literature are as follows. Firstly, the existing body of research predominantly concentrates on the economic effects of corporate ESG scores [4, 7], with little attention given to the study of its driving factors. In contrast, this paper takes the initiative to investigate the driving factors from a macro-institutional perspective, incorporating the social credit environment as an informal institution into the research framework of factors driving corporate ESG scores. Through empirical testing, it is found that an improvement in the credit environment contributes to enhancing company ESG performance. This not only enriches the informal institutional perspective of influencing factors on corporate ESG but also provides new empirical evidence for the field of research on factors affecting corporate ESG performance. Secondly, prior researches mainly concentrate on the influence of social credit on economic behaviors such as corporate transaction costs [26], social responsibility [27], environmental responsibility [25], and corporate digital transformation [28], with insufficient attention given to ESG levels. Therefore, this paper approaches from the angle of corporate sustainable development, exploring whether social credit research influences corporate ESG scores, thereby enriching the microeconomic effects research on the social credit environment. Finally, this paper explores how social credit system construction model cities impact corporate ESG performance through green technological innovation, ethical and moral culture, and optimization of the business environment. It opens the "mechanism black box" of how the improvement in the credit environment promotes the enhancement of corporate ESG performance, contributing to the study of institutionalized social trust amidst China’s social transformation and laying a theoretical foundation for raising the level of social trust.

## 2 Theoretical analysis and research assumptions

### 2.1 Policy background

To standardize the market economy order and enhance the market credit environment, the Chinese government initiated the path of improving the social credit environment in 2003. This involved the integration of national basic databases and financial information databases to effectively document credit behavior and generate credit reports, thereby establishing a groundwork for corporate credit rating. A significant milestone in this endeavor occurred in 2014 when China released the "Outline of the Plan for Building a Social Credit System (2014–2020)" providing further clarity on the overall framework of the social credit system. This period marked the comprehensive development stage of the system’s construction. Notably, in July 2015 and April 2016 alone, China successively approved 43 pilot cities for the construction of the social credit system. This initiative aimed to reduce transaction costs and guard against systemic financial risks. In December 2020, the Chinese government further defined the scope of credit information, perfected the mechanism for punishment and restoration of trustworthiness, and propelled the social credit environment towards a higher level.

The pilot cities have implemented a modernized information collection and data analysis system, enhancing the credit records of market entities. By building upon the collection and integration of fundamental credit information for individuals and businesses, these cities have conducted comprehensive and standardized credit assessments encompassing all market entities. Concurrently, measures have been taken to enhance transparency in credit assessments. This involves publicly ranking corporate social credit through methods such as credit ratings and "red-black lists," thereby making this information accessible to the public and advancing the transparency of credit evaluation. Moreover, the pilot cities have instituted a credit-based regulatory mechanism. This mechanism reinforces collaborative disciplinary actions against seriously untrustworthy enterprises, institutions, and individuals through periodic rectification, joint penalties, and restrictions on market or industry access. In essence, the public disclosure of corporate credit information directly impacts corporate reputation, increasing the value of maintaining good faith and the cost of dishonesty. Consequently, businesses are incentivized to better fulfill their social responsibilities, thereby contributing to sustainable development.

### 2.2 Research hypothesis

From a broad perspective, corporate ESG is an extended manifestation of the conceptual framework of CSR investment. According to new institutional economics, the level of development and behavioral decisions of corporate organizations largely depend on the institutional environment in which they are located [29]. Social credit is not only a social norm, but also an important part of the external institutional environment of the corporate, which is manifested in the moral norms that constrain people’s behavior. It has been found that optimization of the social credit environment encourages corporate decision-makers to adopt more ethical corporate behaviors [30, 31], and that such ethical behaviors enhance the organizational ethical culture, promote long-term orientation of decision-making, and exert ethical responsibility initiatives about the natural environment, consumers, suppliers, governance, and society [32]. In addition, social credit improvement can also directly contribute to the level of business credit environment [33, 34], enhance the level of mutual trust among firms, reduce information asymmetry and communication costs, and indirectly enhance corporate concern for social reputation, which in turn acts on corporate ESG performance [35]. Specifically, this article argues that the pilot implementation of the social credit system can enhance the corporate ESG scores through the following three mechanisms.

Green technology innovation effect. As a highly uncertain behavior, corporate technological innovation is often affected by the external environment of the corporation. The enhancement of the credit environment cultivates a more equitable and efficient market atmosphere for enterprises. This, in turn, nurtures intrinsic motivation for innovation by diminishing transaction costs and intensifying market competition. Under the background of high levels of social credit, corporate managers place more attention on maintaining corporate image and reputation when making decisions [36], so green technology innovation is a good choice. Firstly, as the level of social credit improves, corporations’ reputation and access to financing channels increase, enabling them to obtain financing from financial institutions at a lower cost, thus effectively reducing the financing constraints faced by corporations [8]. Secondly, the establishment and refinement of social credit system means that opportunism is replaced by trust, corporations do not have to worry about the risk of corporate green patents being seized [33], and the subjective willingness of green innovation is enhanced. Finally, green technology innovation is an important way approach for corporations to fulfill environmental responsibility and obtain high-quality corporate development [37], and the use of their green patents usually reduces corporate pollutant emissions, which is conducive to enhancing corporate image. Green technological innovation by corporations not only promotes environmental protection and social responsibility [38], but also is an expression of corporate fulfillment of social and employee responsibilities [39]. As a pivotal component of sustainable strategies, the innovative endeavors in green technologies by enterprises frequently garner heightened attention from investors and policy support. This assistance aids companies in attaining sustained competitive advantages [40]. In general, the higher the sustainability of policies, the more robust the green technological innovation investments of enterprises [19, 41]. This trend becomes more pronounced in driving the ESG performance of enterprises. Therefore, the establishment of a social credit system can contribute to the improvement of local enterprise ESG performance by fostering innovation in green technologies within businesses.

Ethical and moral culture effects. Wu et al. (2015) [32] identified a notable positive correlation between corporate ethical and moral culture and corporate social responsibility scores. The establishment of the social credit system, operating as an informal institutional mechanism, elevates the ethical standards of corporate employees and enriches the organizational culture. This reinforcement amplifies the emphasis on fulfilling corporate social responsibility for sustainable development. Consequently, it contributes to an enhancement in the overall ESG scores of the enterprise. From the individual perspective, credit, viewed as a positive moral manifestation [23, 42], broadens the scope of behavioral considerations and motivates individuals to engage in more ethical conduct [43]. Consequently, an elevation in social credit levels can notably restrain individual selfishness, thereby reinforcing a sense of responsibility and the likelihood of assuming responsibility [44]. From an organizational perspective, grounded in social system theory, social constraints impact each member within a system [45]. In other words, normative constraints such as credit exert influence throughout the organization via members’ social networks. Therefore, the construction of a social credit system will foster ethical norms within corporate culture, promoting an altruistic orientation and a proclivity for responsible conduct in dealings with the environment, consumers, society, employees, and shareholders. In other words, cities with good credit environments are more capable of stimulating altruism and responsible behavior in local corporate organizations, which in turn motivates corporations to participate in environmental protection, social governance, and other sustainable activities. Therefore, pilot cities implementing the social credit system enhance the ESG scores of companies by advancing the development of corporate ethical and moral culture.

Business environment optimization effect. It has been noted that corporate managers in business strategies usually allocate more of their limited funds to high-return sectors rather than to environmental protection or social responsibility [46]. Institutional theory emphasizes that the organization’s external environment influences the flow of corporate capital allocation [29]. Among the many factors of the corporate external environment, institutional external factors such as rules, laws, regulations, norms, or culture are more important in determining the development orientation of a corporation compared to the degree of market competition [22]. Diverging from mandatory environmental regulations such as legal statutes, the social credit environment establishes intangible soft constraints on corporate behavior, diminishing instances of misconduct. Consequently, this optimization contributes to a business-friendly and transparent operating environment. On the one hand, the optimization of the business environment can better protect the intellectual property rights of enterprises and directly promote the investment of enterprises in product quality [47]. At the same time, the openness of trade brought about by it will inevitably give rise to the entry of foreign-funded enterprises and increase the competitiveness of the market [21], which will compel companies to upgrade production and product quality, thus better safeguarding the relationship between the corporations and the consumers; On the other hand, the optimization of business environment brought about by the perfection of the credit system is conducive to reducing corporate institutional costs and improving corporate governance, thus alleviating principal-agent conflicts [48]. This means that the interests of corporate stakeholders converge and management is more likely to uphold the responsibilities of other stakeholders [49], thus enhancing Perfection of the credit system. At the same time, the soft constraints of the social credit environment drive decision-makers to allocate more resources towards environmental protection and cultivating corporate reputation, aiming to uphold a responsible and reliable public image for the enterprise [36]. This subsequently boosts corporations’ willingness to sustainability of corporate development, fostering the enhancement of corporate ESG scores. Therefore, the refinement of the social credit system can contribute to the enhancement of corporate ESG scores by optimizing the business environment.

In summary, this study proposes the research hypothesis:

H: The construction of a social credit system contributes to the enhancement of corporate ESG performance.

## 3 Research design

### 3.1 Sample description and data sources

This study utilizes data from Chinese companies listed on the Shanghai and Shenzhen A-shares markets for the years 2010 to 2020 as its research sample. Following the common practices in existing research, the research sample undergoes the following treatments: firstly, the exclusion of samples with abnormal trading statuses, such as ST and PT; secondly, the removal of data from companies in the insurance and financial industries; thirdly, the deletion of samples with missing key data. Ultimately, a total of 25,073 firm-year observations are obtained. To ensure the reliability of the benchmark regression results and mitigate the potential impact of individual outliers, a two-sided 1% winsorization is applied to all continuous variables in the model. Regarding data sources, corporate ESG performance is obtained from the official website of HuaZheng and manually compiled. Other data are sourced from the CSMAR database. The list of 43 pilot cities and corresponding time periods can be obtained from the official website of the National Development and Reform Commission of China.

### 3.2 Definition of variables

1. Dependent Variable. Numerous global ESG rating agencies have been dedicated to evaluating corporate ESG performance; however, a universally recognized and standardized rating criterion has yet to be established. HuaZheng’s ESG rating stands out as the most comprehensive and data-rich assessment of corporate ESG performance in China. This rating incorporates a multitude of indicators relevant to China’s current development needs, rendering it more scientific and representative [17, 50]. Notably, it not only assigns ESG grades to listed companies (C-A) but also provides detailed scores ranging from 0 to 100. In light of this, our study opts for the more detailed HuaZheng ESG scores as a proxy variable for ESG performance. Furthermore, following the common practice in existing research, the ESG performance of companies is measured by assigning scores from 1 to 9 to each of the nine levels in the HuaZheng ESG rating, used for robustness testing.
2. Independent Variable. Did_scsc, serves as the core explanatory variable in this study. It is a dummy variable used to indicate whether a company is affected by the construction of the social credit system (SCSC). If the company is located in a city on the pilot list, Did_scsc is assigned a value of 1 for the year it is included in the pilot program and subsequent years; otherwise, it is set to 0.
3. Control Variables. Building on the practices of previous studies [20, 51], we control for various firm-level variables that may influence corporate ESG. Specifically, the company-specific characteristics encompass firm size (Size), leverage (Lev), profitability (Roa), corporate age (Age), and corporate growth opportunities (TobinQ). Governance variables include whether there is a dual role of CEO and chairman (Duality), board size (Board), and the proportion of independent directors (Indep). Finally, our analysis incorporates year-fixed effects and firm-fixed effects. The variable names and corresponding definitions are presented in Table 1.

**Table 1:** Variable definitions.

Variable	Definition
ESG	HuaZheng Corporate ESG Scores
Did_scsc	Whether a company is influenced by the policy of social credit system construction is indicated by a binary variable. If the company is affected, it is set to 1; otherwise, it is set to 0
Size	Natural logarithm of year-end total assets
Lev	Total assets to total liabilities ratio
Roa	Net profit to total assets ratio
Duality	Dummy variable that combines two positions, if yes, assign 1; otherwise, assign 0
TobinQ	Market value to total assets ratio
Age	Natural logarithm of the enterprise’s age plus 1
Board	Natural logarithm of board size
Indep	The proportion of independent directors to total board members

### 3.3 Model design

To validate the hypotheses established earlier in the study, we adopt the 2015 and 2016 batches of social credit system pilots as quasi-natural experiments. Drawing inspiration from the methodologies of Cui et al. (2023) [25], Qiu et al. (2023) [28], and Sun et al. (2023) [52], we construct the following multi-period difference-in-differences model for empirical testing.


$${\mathrm{ESG}}_{\mathrm{i,t}}=\alpha_{0}+\alpha_{1}{\mathrm{Did}\_\mathrm{scsc}}_{\mathrm{i,t}}+\sum \beta X_{\mathrm{i,t}}+\lambda_{t}+\mu_{i}+\varepsilon_{\mathrm{i,t}}$$ (1)

In Model (1), *i* represent firms, and *t* represent years. *ESG*_*i*,*t*_ denotes the ESG score of the firm. The explanatory variable *Did*_*scsc*_*i*,*t*_ measuring whether the city has the SCSC. *X* encompasses a series of control variables that may influence corporate ESG performance. λ_t_ denotes year-fixed effects, *μ*_*i*_ represents firm-fixed effects, *α*_*0*_ and *ε*_*i*,*t*_ are the intercept and error term, respectively. Furthermore, to address autocorrelation and heteroscedasticity issues over time for different firms, wo cluster the standard errors at the corporate level. This study places particular emphasis on the coefficient *α*_*1*_ of *Did*_*scsc*_*i*,*t*_. A positive and significant *α*_*1*_ would imply that the SCSC is conducive to enhancing firms’ ESG scores.

## 4 Regression results

### 4.1 Descriptive statistics

Table 2 shows the descriptive statistics for the primary variables. The mean of ESG is 73.029, displaying a positive trend in the overall ESG scores of Chinese-listed companies. With a minimum of 56.94, a maximum of 84.25, and a standard deviation of 5.29, there is notable variability in performance levels among the sampled firms. The mean value of Did_scsc is 0.307, suggesting that approximately 30.7% of the sample is affected by the SCSC pilot cities. Among the control variables, the mean of Duality is 0.267, signifying that 26.7% of the sample companies have the same individual serving as both the chairman and CEO. Additionally, the descriptive statistics of the control variables in this study align with existing research and adhere to common knowledge.

**Table 2:** Descriptive statistics of main variables.

Variable name	Observation	Mean	S. D.	Min	Max
ESG	25073	73.029	5.29	56.94	84.25
Did_scsc	25073	0.307	0.461	0	1
Size	25073	22.187	1.167	20.42	24.664
Lev	25073	0.428	0.205	0.055	0.899
Roa	25073	0.037	0.062	-.288	.192
Duality	25073	0.267	0.442	0	1
TobinQ	25073	2.05	1.322	0.861	8.804
Age	25073	2.166	0.763	0.693	3.434
Board	25073	2.134	0.199	1.099	2.89
Indep	25073	37.459	5.543	16.67	80

### 4.2 Benchmark regression results

Table 3 reports the baseline regression results for Model (1) based on the stepwise regression approach. The mixed-OLS regression results without any control variables are shown in Table (1). Column (2) reports the outcomes with fixed effects for both years and firms. Column (3) reports the result with further controls for variables that may influence corporate ESG scores based on the specifications in Column (2). It is observed that, the regression coefficient of Did_scsc in the mixed OLS model is 0.5951 and statistically significant at the 1% level. This suggests that the SCSC significantly promotes the improvement of corporate ESG scores, providing preliminary support for the hypothesis of this study. The results in Column (3) show that the regression coefficient of Did_scsc is 0.4104, slightly higher in absolute value compared to Column (2), and significant at the 1% level. This implies that, compared to non-pilot cities, the pilots of SCSC have a positive impact on the local corporate ESG scores. In other words, the SCSC indeed yields positive social effects by enhancing the credit environment. This development can effectively elevate corporate ESG performance, assuming the premise stated in this article holds true.

**Table 3:** Benchmarking results.

	(1)	(2)	(3)
	ESG	ESG	ESG
Did_scsc	0.5951***	0.3575**	0.4104***
	(8.28)	(2.35)	(2.74)
Size			1.0938***
			(8.84)
Lev			-3.7362***
			(-8.91)
Roa			4.5210***
			(6.02)
Duality			-0.1657
			(-1.35)
TobinQ			0.0104
			(0.28)
Age			-0.9903***
			(-5.17)
Board			0.1328
			(0.32)
Indep			0.0538***
			(4.65)
Constant	72.8384***	72.8935***	49.9310***
	(1,826.91)	(1,569.28)	(17.87)
N	25,413	25,223	25073
Year effect	NO	YES	YES
Firm effect	NO	YES	YES
R^2^	0.003	0.535	0.549
F	68.63	5.522	33.78

Note: *, **, and *** represent significance at the 10%, 5%, and 1% levels, respectively, with the t-values calculated based on enterprise-level clustering-adjusted standard errors, indicated in parentheses, throughout.

One plausible explanation for this phenomenon is that the amelioration of the credit environment effectively extends the temporal orientation of corporate decision-making, consequently enhancing corporate ESG performance. Primarily, the social credit system introduces mechanisms like environmental information monitoring and red-black lists, prompting enterprises to partake in green innovation activities to fulfill their environmental responsibilities. Furthermore, the elevation of social credit can induce companies to fortify the development of ethical cultures, fostering robust corporate behavioral norms. In such an environment, companies are more inclined to adhere to laws and regulations, uphold human rights, prioritize social well-being, and adopt responsible business practices. Lastly, the enhancement in the social credit environment is conducive to cultivating a more transparent, fair, and trustworthy business milieu, thereby amplifying the commercial reputation and corporate value of reliable enterprises. In a positive business atmosphere, companies are more motivated to embrace sustainable business models, emphasizing environmental protection, employee well-being, and social responsibility, ultimately resulting in exceptional ESG performance.

### 4.3 Parallel trends test

**Fig 1:**
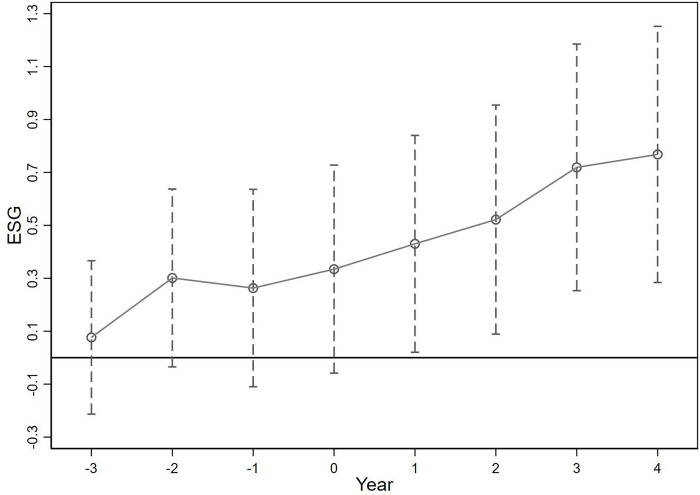
Parallel trend test.

To examine whether local enterprises in social credit system construction pilot cities (treatment group) and enterprises without social credit system construction (control group) had similar trend changes before the policy implementation, we set the policy implementation year as year 0 and use four pre-policy implementation dummy variables (pre4, pre3, pre2, and pre1), as well as four post-policy implementation dummy variables (post1, post2, post3, and post4) with the current year as the policy implementation year. We replace the Did_scsc in Model (1) with these virtual variables and conduct a regression analysis, along with plotting parallel trend graphs. The anticipated result is that the coefficients of the four pre-implementation virtual variables are all statistically insignificant, satisfying the parallel trends test. Fig 1 presents the regression coefficient magnitudes and ranges for each virtual variable. The regression coefficients for the pre-implementation virtual variables before policy implementation all encompass zero, indicating a successful parallel trends test. In contrast, the ranges of regression coefficients for the post-implementation virtual variables do not include zero. Thus, there is a valid reason to consider social credit system pilot cities as an external shock event.

### 4.4 Parallel trends test

#### 4.4.1 Placebo test

**Fig 2:**
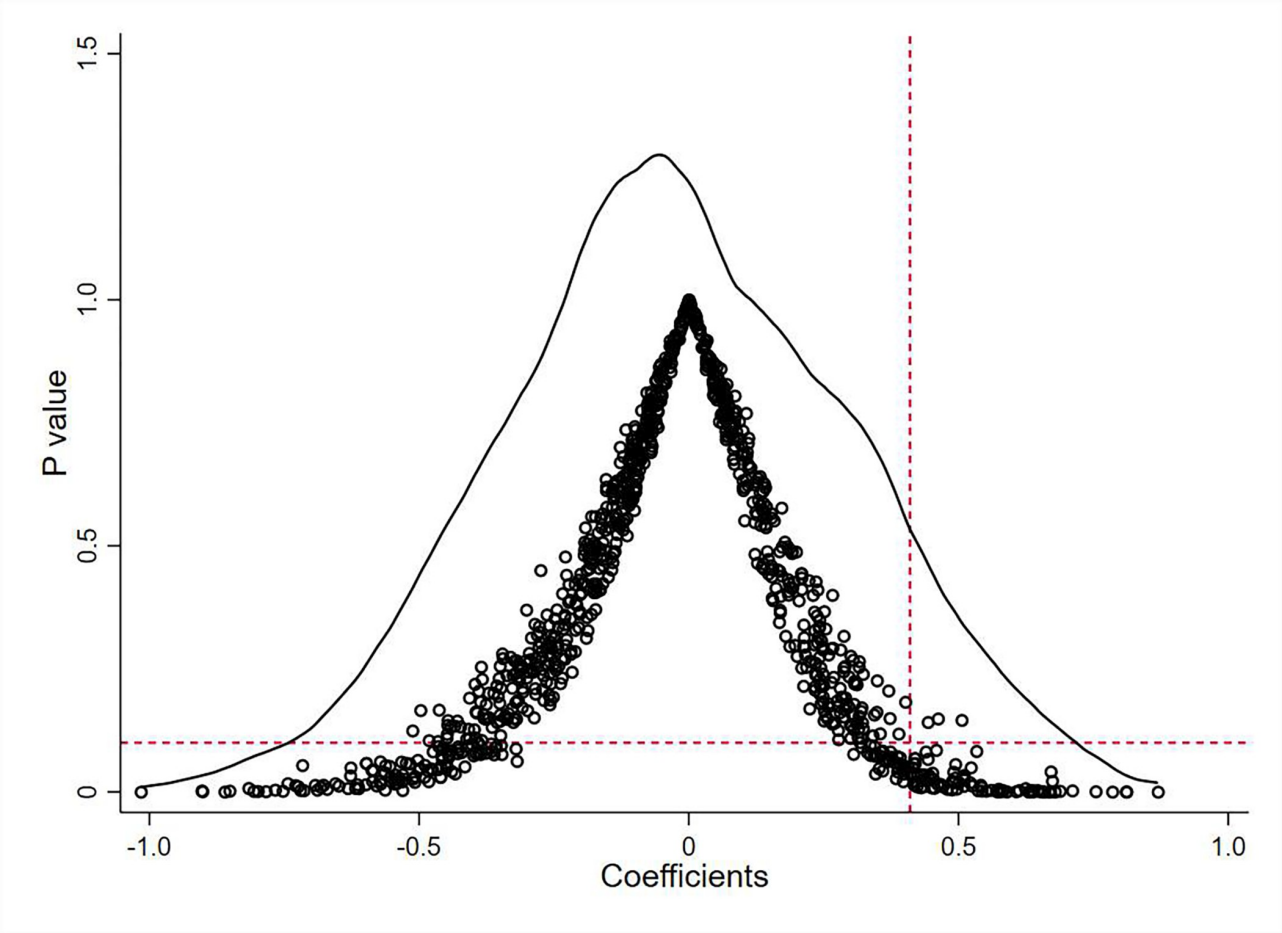
Placebo test.

To control for the impact of unobservable variables beyond the scope of years and companies on the baseline regression results, this study conducted a placebo test from the perspective of a random sample. Specifically, based on the pilot project’s generated time and the number of experiments, social credit system construction cities were randomly simulated, and the regression was re-performed. We repeated this process 1000 times. Fig 2 displays the distribution of estimated coefficients and corresponding p-values from the random sampling placebo test. It is obvious that the estimated coefficient distribution for randomly generated social credit system construction pilots is centered around zero, significantly lower than the estimated coefficients from the baseline regression. Additionally, the majority of p-values do not pass the significance test, aligning with the expected outcomes of the placebo test. This indicates that the conclusion that constructing a social credit system enhances corporate ESG performance is not influenced by other unobservable factors.

#### 4.4.2 PSM-DID test

Given the relatively low proportion of samples from social credit system construction pilot cities, and to minimize potential sample selection bias, following the approach of Qiao and Wen. (2023) [53], this study employs propensity score matching (PSM) to access the reliability of the baseline regression results. Firstly, we employed the nearest neighbor matching method to construct a Probit model for whether a company belongs to the social system reform pilot. Using a 1:1 with replacement method, we matched each enterprise in a pilot city with a non-pilot region enterprise that was most similar in terms of company characteristics. Subsequently, we conduct regression analysis using the matched sample. Secondly, we further conducted an endogeneity test using a non-parametric kernel matching method. In both matching methods, the matching variables are consistent with the control variables in the baseline model. Table 4 provides the regression results for the matched sample, revealing that the regression coefficients of Did_scsc are consistently and significantly positive. This indicates that even after overcoming potential sample selection bias, the SCSC continues to effectively enhance corporate ESG scores, aligning with the regression results discussed earlier.

**Table 4:** PSM—DID results.

	(1)	(2)
	ESG	ESG
Did_scsc	0.3275**	0.4106***
	(2.08)	(2.74)
Size	1.0791***	1.0956***
	(7.68)	(8.85)
Lev	-3.4519***	-3.7288***
	(-7.26)	(-8.88)
Roa	5.3788***	4.5336***
	(6.38)	(6.03)
Duality	-0.1221	-0.1633
	(-0.91)	(-1.33)
TobinQ	-0.0275	0.0107
	(-0.68)	(0.28)
Age	-0.9285***	-0.9957***
	(-4.41)	(-5.19)
Board	0.2609	0.1325
	(0.57)	(0.32)
Indep	0.0583***	0.0542***
	(4.53)	(4.68)
Constant	49.6718***	49.8845***
	(15.49)	(17.82)
N	19,556	24,874
Year effect	YES	YES
Firm effect	YES	YES
R^2^	0.551	0.549
F	27.44	33.82

#### 4.4.3 Excluding the impact of other policies

Considering the influence of various policies during the sample period and aiming to minimize the potential impact of these policies on the reliability of the baseline results, this study excludes the effects of smart city initiatives, Broadband China pilot cities, and low-carbon city initiatives. To assess the net effect of constructing the social credit system on enhancing company ESG, the regression results are presented in Table 5. In Columns (1) through (3), the influence of SCSC on corporate ESG is documented, with controls for the interference of Broadband China policy, smart city policy, and low-carbon city policy. It is evident that the estimated coefficients of Did_scsc are significantly positive at the 1% level, indicating that the improvement in the credit environment brought about by the SCSC contributes to the enhancement of company ESG performance. This affirms the reliability of the baseline results.

**Table 5:** Excluding other policy effects.

	(1)	(2)	(3)
	ESG	ESG	ESG
Did_scsc	0.4426***	0.4188***	0.3949***
	(2.92)	(2.79)	(2.63)
Broadband China city	-0.1097		
	(-0.76)		
Smart city		0.3939**	
		(2.50)	
Low-carbon city			0.2516*
			(1.69)
Size	1.0948***	1.0982***	1.0948***
	(8.85)	(8.88)	(8.85)
Lev	-3.7374***	-3.7441***	-3.7429***
	(-8.90)	(-8.94)	(-8.93)
Roa	4.5150***	4.5125***	4.4991***
	(6.01)	(6.01)	(6.00)
Duality	-0.1642	-0.1643	-0.1656
	(-1.34)	(-1.34)	(-1.35)
TobinQ	0.0111	0.0112	0.0098
	(0.30)	(0.30)	(0.26)
Age	-0.9937***	-0.9757***	-0.9883***
	(-5.18)	(-5.10)	(-5.16)
Board	0.1279	0.1109	0.1361
	(0.31)	(0.27)	(0.33)
Indep	0.0537***	0.0534***	0.0540***
	(4.64)	(4.63)	(4.66)
Constant	49.9783***	49.7318***	49.7641***
	(17.88)	(17.80)	(17.80)
N	25,073	25,073	25,073
Year effect	YES	YES	YES
Firm effect	YES	YES	YES
R^2^	0.549	0.549	0.549
F	30.61	30.92	30.47

#### 4.4.4 Other robustness tests

To further ensure the reliability of the baseline regression results and minimize estimation errors, this study conducts robustness checks from four perspectives, with the findings presented in Table 6. Firstly, by adjusting the model’s clustering hierarchy, we employ a more stringent city-level clustering for the robust standard errors of the baseline model. The results in the first column indicate that Did_scsc remains significantly positive. This suggests that even under stricter clustering standards, SCSC continues to effectively enhance corporate ESG performance. Secondly, to control for the potential influence of measurement errors in key variables on the baseline results, we adopt the approach of Hu et al. (2023) [8] and Wang et al. (2023) [14]. We replace the dependent variable with the HuaZheng ESG rating assigned values of 1–9 as a new proxy variable for corporate ESG scores and re-conduct the regression. The results in column (2) show that even after this substitution, the coefficient for Did_scsc remains significantly positive. Thirdly, to address the possible impact of macro-level city variables on empirical results, we include city-fixed effects in the existing model. The results in column (3) demonstrate a significant positive correlation between cities implementing the social credit system and corporate ESG scores. Finally, considering the dynamic effects of corporate ESG performance and the potential impact of residual sequence correlation on model results, following the approach of [25], we introduce the lag term of corporate ESG performance (L.ESG) as a control variable in the model (1) and re-conduct the regression, as shown in column (4). Notably, accounting for lag effects, the credit environment improvement brought about by the SCSC contributes to better local corporate ESG performance.

**Table 6:** Other robustness tests.

	(1)	(2)	(3)	(4)
	ESG	ESG_new	ESG	ESG
Did_scsc	0.4104**	0.0786**	0.3803**	0.2432**
	(2.07)	(2.57)	(2.50)	(2.11)
L.ESG				0.3432***
				(37.78)
Size	1.0938***	0.2193***	1.1046***	0.7751***
	(8.86)	(8.71)	(8.79)	(7.76)
Lev	-3.7362***	-0.7460***	-3.4650***	-2.8733***
	(-8.48)	(-8.68)	(-8.63)	(-7.72)
Roa	4.5210***	0.8725***	4.5949***	4.3154***
	(5.56)	(5.65)	(6.03)	(6.19)
Duality	-0.1657	-0.0505**	-0.1558	-0.0474
	(-1.27)	(-1.99)	(-1.26)	(-0.44)
TobinQ	0.0104	-0.0061	0.0150	-0.0166
	(0.19)	(-0.78)	(0.40)	(-0.48)
Age	-0.9903***	-0.1948***	-0.9960***	-0.8195***
	(-4.27)	(-4.95)	(-5.13)	(-3.70)
Board	0.1328	0.0213	0.1349	-0.0155
	(0.34)	(0.25)	(0.32)	(-0.04)
Indep	0.0538***	0.0108***	0.0535***	0.0428***
	(3.99)	(4.57)	(4.60)	(4.35)
Constant	49.9366***	-0.5139	49.6733***	32.0855***
	(17.04)	(-0.90)	(17.52)	(14.04)
N	25,073	24,938	25,073	21,103
Year effect	YES	YES	YES	YES
Firm effect	YES	YES	YES	YES
City fixed effect	NO	NO	Yes	NO
R^2^	0.549	0.504	0.546	0.617
F	39.04	32.68	32.28	175.9

### 4.5 Heterogeneity tests

#### 4.5.1 Property rights heterogeneity

To assess whether the improvement of the social credit environment differentially impacts the ESG scores of companies with varying property rights, this study categorizes companies into state-owned (SOEs) and non-state-owned enterprises (Non-SOEs) based on the nature of controlling shareholders. Separate regressions are conducted for each sub-sample. The findings in columns (1) and (2) of Table 7 reveal that in Non-SOEs, the estimated coefficient of Did_scsc is not statistically significant. This contrasts with the statistically significant positive impact coefficient observed in SOEs. These findings suggest that the motivating effect of the credit environment improvement, brought about by the social credit system, on ESG scores of enterprises varies across different property rights. This variability may be attributed to the distinctive political environment in China. SOEs have clear political affiliations, and their day-to-day operations and strategic decisions typically involve regulatory oversight from government agencies. Hence, they are more inclined to abide by laws, fulfill corporate responsibilities, and shape an exemplary image [17]. On the contrary, Non-SOEs do not face the same constraints in molding their corporate image [25], resulting in a relatively weaker intrinsic motivation to enhance ESG responsibilities. Consequently, the impact of SCSC on the improvement of ESG scores is more pronounced in SOEs.

**Table 7:** Heterogeneity regression results.

	(1)	(2)	(3)	(4)
	SOEs	Non-SOEs	High-IS	Low-IS
	ESG	ESG	ESG	ESG
Did_scsc	0.7292***	0.1419	0.4658**	0.3218
	(3.35)	(0.69)	(2.41)	(1.31)
Size	1.0555***	1.2835***	1.2170***	1.3005***
	(5.33)	(7.78)	(7.02)	(7.16)
Lev	-3.3732***	-3.7846***	-3.5861***	-3.7683***
	(-4.98)	(-6.95)	(-5.78)	(-6.45)
Roa	2.0081	4.0346***	3.3763***	3.3342***
	(1.36)	(4.51)	(2.87)	(3.36)
Duality	-0.1982	-0.1018	-0.0394	-0.1835
	(-0.93)	(-0.66)	(-0.22)	(-1.03)
TobinQ	-0.0469	0.0564	0.0181	0.0274
	(-0.75)	(1.21)	(0.33)	(0.45)
Age	1.6097***	-0.5786**	-0.0081	-0.8529***
	(3.80)	(-2.34)	(-0.02)	(-3.01)
Board	0.5628	-0.3826	0.0757	0.3714
	(0.97)	(-0.67)	(0.13)	(0.58)
Indep	0.0697***	0.0341**	0.0669***	0.0561***
	(4.40)	(2.05)	(4.39)	(3.15)
Constant	43.1621***	46.3806***	44.6683***	44.2444***
	(9.42)	(12.43)	(10.96)	(10.69)
N	9,572	15,036	12,785	12,288
Year effect	YES	YES	YES	YES
Firm effect	YES	YES	YES	YES
R^2^	0.593	0.534	0.605	0.528
F	11.72	18.88	13.82	15.94

#### 4.5.2 Institutional shareholding heterogeneity

Dyck et al. (2019) [54] point out that firms with high institutional shareholding ratios tend to place significant value on reputation and harbor subjective incentives to enhance their corporate environmental and social performance, leading to superior E&S performance. This propensity is rooted in the fact that, in comparison to individual investors, institutional investors possess specialized technical expertise and maintain a long-term perspective. They exhibit heightened concern for the sustained, long-term development of corporations and recognize the value derived from the company’s commitment to environmental protection and governance responsibility [10, 31]. Consequently, institutional investors have inherent incentives to actively engage in corporate behavioral decisions, encouraging corporations to allocate resources towards environmental protection and the enhancement of corporate image. Moreover, companies with elevated institutional ownership are more likely to curb managerial short-sightedness, fostering a greater willingness and quality in environmental information disclosure [31], and consequently promoting a long-term orientation in managerial decision-making. Finally, companies with high institutional ownership typically enjoy the advantage of obtaining financing at lower costs, thereby increasing their willingness to enhance non-financial indicators [55]. In contrast, companies with lower institutional ownership, constrained by short-sightedness and financial limitations, their subjective willingness to engage in sustainable practices such as fulfilling environmental and social responsibilities, as well as improving corporate governance is inhibited. Therefore, the SCSC may yield differing impacts on the ESG performance of companies, contingent upon their ownership ratios.

Given this, this paper classifies companies with institutional ownership ratios exceeding the average value as the high institutional shareholding sub-sample (High-IS), while the rest are classified as the low institutional shareholding sub-sample (Low-IS). Sub-sample regressions are subsequently conducted. The results in Table 7, columns (3) and (4), show that the coefficient of Did_scsc is 0.4658 for companies with high institutional ownership ratios, and it is significant at the 5% level. In contrast, in companies with low institutional ownership ratios, although the coefficient of Did_scsc is positive, it fails to reach statistical significance. This implies that the pilots of SCSC significantly promotes the enhancement of ESG performance in companies characterized by high institutional ownership ratios.

## 5 Further testing

The preceding analysis has already validated that the construction of the social credit system, leading to an improvement in the credit environment, can significantly enhance company ESG scores. Building upon the theoretical analysis in the previous sections, mechanisms such as green technological innovation, ethical and moral culture, and business environment optimization may be at play. To examine the existence of these mechanisms, we construct the following empirical mediation test model for verification.


$$M_{\mathrm{i,t}}=\alpha_{0}+\alpha_{1}{\mathrm{Did}\_\mathrm{scsc}}_{\mathrm{i,t}}+\sum \beta X_{\mathrm{i,t}}+\lambda_{t}+\mu_{i}+\varepsilon_{\mathrm{i,t}}$$ (2)

$${\mathrm{ESG}}_{\mathrm{i,t}}=\alpha_{0}+\gamma M_{\mathrm{i,t}}+\alpha_{1}\mathrm{Di}{d\_\mathrm{scsc}}_{\mathrm{i,t}}+\sum \beta X_{\mathrm{i,t}}+\lambda_{t}+\mu_{i}+\varepsilon_{\mathrm{i,t}}$$ (3)

Where *M*_*i*,*t*_ represents the mediating variables, including green technological innovation (Green), corporate ethical and moral culture (Ethics), and regional business environment (Citytrust). To be more specific, green technological innovation (Green) is represented by the natural logarithm of the sum of the number of patent applications for green inventions and green utility models by the company, plus one. The data is sourced from the CNRDS database. Referring to Wu et al. (2015) [32], corporate ethical and moral culture (Ethics) is quantified by utilizing the CESG repository within the CNRDS database. The metrics involve the arithmetic computation and characterization of virtual variables about charity, volunteer activities, and advantages in social dispute resolution. Higher arithmetic values are indicative of a more robust corporate ethical and moral culture, underscored by a heightened altruistic ambiance and a proclivity towards pro-social conduct. The regional business environment (Citytrust) is expressed using the Business Credit Environment Index of Chinese Cities published by the China Academy of Management Sciences. Other variables and definitions are consistent with model (1).

Table 8 reports the regression results for the mechanism tests. Columns (1) and (2) represent the mechanism of green technological innovation, where the regression coefficient for the Did_scsc is 0.0630. This indicates that the pilots of SCSC can significantly promote the output of green patents by companies. In column (2), the coefficient for Did_scsc is 0.3873, significant at the 1% level. What’s more, compared to the model without the Green variable, the coefficient has slightly decreased, suggesting that Green is a partial mediator. This confirms that green technological innovation is one of the mechanisms through which the pilot cities of the social credit system drives the enhancement of corporate ESG scores. We further categorize corporate green innovation into green innovation quality and green innovation quantity. Findings reveal that the mechanism of green technological innovation is primarily reflected in the quality of green innovation, while the path of the quantity of corporate green innovation is not statistically significant.

**Table 8:** Mechanism test regression results.

	(1)	(2)	(3)	(4)	(5)	(6)
	Green	ESG	Ethics	ESG	Citytrust	ESG
Did_scsc	0.0630**	0.3873***	0.1697***	0.3224**	1.9450***	0.3823**
	(2.23)	(2.60)	(4.14)	(2.21)	(8.05)	(2.54)
Green		0.3663***				
		(6.98)				
Ethics				0.5184***		
				(12.33)		
Citytrust						0.0145*
						(1.68)
Size	0.3486***	0.9661***	0.2830***	0.9471***	0.1577	1.0915***
	(12.48)	(7.76)	(7.28)	(7.85)	(0.82)	(8.82)
Lev	0.0765	-3.7642***	-0.2200**	-3.6221***	0.0982	-3.7376***
	(1.00)	(-9.04)	(-2.21)	(-8.87)	(0.10)	(-8.91)
Roa	0.0819	4.4910***	0.2042	4.4151***	1.0943	4.5051***
	(0.75)	(6.00)	(1.43)	(5.97)	(1.02)	(6.01)
Duality	0.0152	-0.1712	-0.0163	-0.1572	-0.0135	-0.1655
	(0.71)	(-1.40)	(-0.58)	(-1.30)	(-0.10)	(-1.35)
TobinQ	0.0173***	0.0041	0.0455***	-0.0132	-0.0897*	0.0117
	(2.64)	(0.11)	(4.33)	(-0.36)	(-1.75)	(0.31)
Age	-0.0908**	-0.9571***	-0.4812***	-0.7409***	0.0022	-0.9904***
	(-2.57)	(-5.03)	(-9.43)	(-3.94)	(0.01)	(-5.17)
Board	0.0483	0.1151	0.0255	0.1196	-1.3695*	0.1526
	(0.63)	(0.28)	(0.26)	(0.30)	(-1.77)	(0.37)
Indep	0.0026	0.0529***	-0.0019	0.0548***	-0.0035	0.0539***
	(1.10)	(4.62)	(-0.65)	(4.86)	(-0.20)	(4.65)
Constant	-6.9995***	52.5006***	-4.5466***	52.2938***	68.4416***	48.9469***
	(-11.00)	(18.75)	(-5.11)	(19.22)	(15.19)	(17.19)
N	25,073	25,073	25,073	25,073	25,073	25,073
Year effect	YES	YES	YES	YES	YES	YES
Firm effect	YES	YES	YES	YES	YES	YES
R^2^	0.722	0.551	0.679	0.555	0.965	0.549
F	21.18	36.03	16.94	48.75	10.56	30.56

Columns (3) and (4) show the examination results of the mechanism of corporate ethical and moral culture. Column (3) represent the coefficient for Did_scsc is positive at the 1% level, indicating that the SCSC effectively promotes the development of ethical and moral culture in local companies, enhancing their altruism and sense of responsibility. Adding "Ethics" as a control variable, as shown in column (4), the estimated coefficient is significantly positive. This implies that the strengthening of corporate ethical and moral culture contributes to better ESG performance. These results indicate that the construction of corporate ethical and moral culture is one of the pathways through which the social credit reform affects corporate ESG scores.

Similarly, the estimated coefficients for Did_scsc and Citytrust in columns (5)-(6) are both significantly positive, verifying that the optimization of the business environment is a crucial pathway for the reformation of social credit system to enhance corporate ESG performance.

## 6 Conclusion and policy recommendations

In the era of dual carbon goals and economic transformation, enhancing ESG performance is not only an inevitable requirement for the long-term development of businesses but also holds significant implications for overall economic green transformation and high-quality development. However, existing studies pay insufficient attention to the effects of informal institutions on corporate ESG scores. Thus, we adopt a perspective rooted in informal institutions, integrating the credit environment and ESG scores of companies into a unified economic analysis framework. Utilizing a DID model, this study examines the impact of the improved credit environment resulting from SCSC on corporate ESG scores and further identifies the underlying mechanisms and heterogeneity. The research findings indicate that the SCSC promotes the enhancement of local corporate ESG performance. After multiple robustness checks, this conclusion remains valid, suggesting that the improvement in the credit environment encourages companies to focus more on environmental protection and responsibility. Heterogeneity analysis reveals that, concerning corporate ESG performance, state-owned enterprises and companies with higher institutional ownership benefit more significantly from the improvement in the social credit environment. Mechanism analysis demonstrates that, while optimizing the business environment, the social credit system construction also promotes green technological innovation by companies and the formation of corporate ethical and moral culture, thereby driving the enhancements in company ESG performance.

The conclusions drawn from this study provide policy implications for promoting corporate ESG performance and sustainable development. Firstly, government authorities should enhance the level of the social credit environment. On one hand, the demonstration cities of the social credit system should play a role model, guiding non-demonstration cities to conduct the social credit system. Leveraging the advantages of extensive digital coverage and strong penetrability, efforts should be made to enhance the application of information technology and digitization in social credit system construction, improving the efficiency of collecting, organizing, and evaluating social credit information. On the other hand, intensifying efforts in the construction of the social credit system involves expanding the scope of evaluation to encompass more economic entities within society. Simultaneously, there is a need to enhance the channels for disclosing social credit information and the quality of shared information.

Secondly, efforts should be made to guide corporate investments towards long-term perspectives and the cultivation of corporate ethical and moral culture. Specifically, the government can provide moderate incentives for corporate green innovation activities, particularly those of high-quality green innovation, to encourage companies to proactively strengthen environmental management and protection. Training programs focusing on the ethical and moral aspects of CEOs should be reinforced to foster corporate ethical and moral culture, utilizing soft constraints to guide companies toward more ethical decision-making. Emphasis should be placed on enhancing corporate responsibility in areas such as the environment, consumer protection, and governance, thereby elevating levels of ESG performance.

Additionally, the role of market supervision should be maximized. Institutional investors, often possessing a long-term orientation, tend to promote sustainable corporate development. As a result, they tend to oversee corporate decision-makers to make choices conducive to ESG improvement. Simultaneously, the influential role of media and public external supervision pressure should be harnessed, amplifying the value rewards stemming from improvements in corporate ESG performance.

Lastly, it is crucial to adhere to the principle of precise and targeted policy implementation, avoiding a one-size-fits-all approach. Considering the heterogeneity among companies and the diverse impact of social credit system construction on promoting corporate ESG performance, a tailored classification of enterprises and differential policies should be implemented to enhance policy coverage.

## References

[1] Wang K, Li T, San Z, Gao H. How does corporate ESG performance affect stock liquidity? Evidence from China. Pacific-Basin Finance Journal. 2023;80. doi: 10.1016/j.pacfin.2023.102087

[2] Gillan SL, Koch A, Starks LT. Firms and social responsibility: A review of ESG and CSR research in corporate finance. Journal of Corporate Finance. 2021;66. doi: 10.1016/j.jcorpfin.2021.101889

[3] Weber O., Environmental social and governance reporting in China. Bus Strategy Environ. 2014;23: 303–317.

[4] Aouadi A, Marsat S. Do ESG controversies matter for firm value? Evidence from international data. J Bus Ethics. 2018;151: 1027–1047.

[5] Arvidsson S, Dumay J. Corporate ESG reporting quantity, quality and performance: Where to now for environmental policy and practice? Bus Strategy Environ. 2022;31:1091–1110.

[6] Handayani M. The effect of ESG performance on economic performance in the high profile industry in Indonesia. J Int Bus Econ. 2019;7: 112–121.

[7] Meng T, Yahya MDH, Ashhari ZM, Yu D. ESG performance, investor attention, and company reputation: Threshold model analysis based on panel data from listed companies in China. Heliyon. 2023. doi: 10.1016/j.heliyon.2023.e20974 37876450 PMC10590943

[8] Hu J, Zou Q, Yin Q. Research on the effect of ESG performance on stock price synchronicity: Empirical evidence from China’s capital markets. Financ Res Lett. 2023. doi: 10.1016/j.frl.2023.103847

[9] Meng TC, Da PY, Wei QZ, Qi JW. How does ESG disclosure improve stock liquidity for enterprises—Empirical evidence from China. Environmental Impact Assessment Review. 2023;98. doi: 10.1016/j.eiar.2022.106926

[10] Chen Z, Xie G. ESG disclosure and financial performance: Moderating role of ESG investors. Int Rev Financ Anal. 2022; 83. doi: 10.1016/j.irfa.2022.102291

[11] Su J, Xue L. ESG Performance, Demographic Trend, and Labour Investment Efficiency in China. Appl Econ Lett. 2023;1–7.

[12] Cucari N, Esposito DS, Orlando B. Diversity of board of directors and environmental social governance: Evidence from Italian listed companies. Corp Soc Responsib Environ Manag. 2018;25: 250–266.

[13] Menicucci E, Paolucci G. Board Diversity and ESG Performance: Evidence from the Italian Banking Sector. Sustainability. 2022;14. doi: 10.3390/su142013447

[14] Wang L, Zhang Y, Qi C. Does the CEOs’ hometown identity matter for firms’ environmental, social, and governance (ESG) performance? Environmental Science and Pollution Research. 2023. doi: 10.1007/s11356-023-27349-8 37127738

[15] Drempetic S, Klein C, Zwergel B. The influence of firm size on the ESG score: Corporate sustainability ratings under review. J Bus Ethics. 2020;167: 333–360.

[16] DasGupta R. Financial performance shortfall, ESG controversies, and ESG performance: Evidence from firms around the world. Financ Res Lett. 2022;46. doi: 10.1016/j.frl.2021.102487

[17] Zhu N, Zhou Y, Zhang S, Yan J. Tax incentives and environmental, social, and governance performance: empirical evidence from China. Environmental Science and Pollution Research. 2023;30. doi: 10.1007/s11356-023-26112-3 36881223

[18] Meng L, Zhang Y. Impact of Tax Administration on ESG Performance—A Quasi-Natural Experiment Based on China’s Golden Tax Project III. Sustainability. 2023; 15. doi: 10.3390/su151410946

[19] Zheng J, Khurram MU, Chen L. Can green innovation affect ESG ratings and financial performance? evidence from Chinese GEM listed companies. Sustainability. 2022;14. doi: 10.3390/su14148677

[20] Lei N, Miao Q, Yao X. Does the implementation of green credit policy improve the ESG performance of enterprises? Evidence from a quasi-natural experiment in China. Econ Model. 2023;127. doi: 10.1016/j.econmod.2023.106478

[21] Deng P, Wen J, He W, Chen YE, Wang YP. Capital market opening and ESG performance. Emerging Markets Finance and Trade. 2023;59: 3866–3876. (In Chinese).

[22] Zhilong T, Hafsi T, Wei W. Institutional determinism and political strategies. Bus Soc. 2009;48: 284–325.

[23] Dong W, Han H, Ke Y, Chan KC. Social trust and corporate misconduct: Evidence from China. J Bus Ethics. 2018;151: 539–562.

[24] Kanagaretnam K.; Lee J.; Lim C. Y.; Lobo G. Societal trust and corporate tax avoidance. Review of Accounting Studies. 2018;23: 1588–1628.

[25] Cui X, Ning X, Xu N, Zhang W. Social credit system construction and corporate environmental performance: A quasi-natural experiment from China. Energy & Environment. 2023. doi: 10.1177/0958305X231199153

[26] Su K, Jiang H. Does social trust restrict dual agency costs? Evidence from China. The European Journal of Finance. 2023;29: 278–306.

[27] Chen X, Wan P. Social trust and corporate social responsibility: Evidence from China. Corp Soc Responsib Environ Manag. 2020;27: 485–500.

[28] Qiu BY, Yu M, Zuo JJ. Does the Construction of Social Credit System Promote Enterprise Digital Transformation?A Quasi-natural Experiment Based on the Pilot Reform of Social Credit System. Journal of Shanghai University of Finance and Economics. 2023;25: 92–106. (In Chinese).

[29] DiMaggio PJ, Powell WW. The iron cage revisited: Institutional isomorphism and collective rationality in organizational fields. Am Sociol Rev. 1983;48: 147–160.

[30] Dong W, Ke Y, Li S, Chen X, Wan P. Does social trust restrain excess perk consumption? Evidence from China. International Review of Economics & Finance. 2021;76:1078–1092.

[31] Liu B, Huang W, Chan KC, Chen T. Social trust and internal control extensiveness: Evidence from China. Journal of Accounting and Public Policy. 2022;41: 2801–2826. doi: 10.1016/j.jaccpubpol.2022.106940

[32] Wu LZ, Kwan HK, Yim FH, Chiu RK, He X. CEO ethical leadership and corporate social responsibility: A moderated mediation model. J Bus Ethics. 2015;130: 819–831.

[33] Kale P, Singh H, Perlmutter H. Learning and protection of proprietary assets in strategic alliances: Building relational capital. Strateg Manag J. 2000;21: 217–237.

[34] Xie F, Zhang B, ZhangW. Trust, incomplete contracting, and corporate innovation. Management Science. 2022;68: 3419–3443.

[35] Mooneeapen O, Abhayawansa S, Mamode KN. The influence of the country governance environment on corporate environmental, social and governance (ESG) performance. Sustainability Accounting, Management and Policy Journal. 2022;13: 953–985.

[36] Lindenberg S. Governance seen from a framing point of view: the employment relationship and relational signalling. In: Nooteboom B, Six F, editors. The trust process in organizations: empirical studies of the determinants and the process of trust development. Edward Elgar Publishing; 2003.pp. 37–57.

[37] Acemoglu D, Aghion P, Bursztyn L, Hemous D. The environment and directed technical change. Am Econ Rev. 2012;102: 131–166. doi: 10.1257/aer.102.1.131 26719595 PMC4692283

[38] Xie X, Zhu Q. How to solve the problem of “harmonious symbiosis” in corporate green innovation practice. Management World. 2021;37: 128–149. (In Chinese).

[39] Li W, Pang W. The impact of digital inclusive finance on corporate ESG performance: based on the perspective of corporate green technology innovation. Environmental Science and Pollution Research. 2023;30: 65314–65327. doi: 10.1007/s11356-023-27057-3 37084053 PMC10119816

[40] Huang X, Hu Z, Fu C, Yu D. The impact mechanism of green innovation strategy on corporate performance: Based on the mediating effect of green dynamic capabilities. Sci Technol Prog Policy. 2015;32: 104–109.

[41] Saunila M, Ukko J, Rantala T. Sustainability as a driver of green innovation investment and exploitation. J Clean Prod. 2018;179: 631–641.

[42] Hu S, Yu Y, Fei Q. Social credit and patent quality: Evidence from China. J Asian Econ. 2023;84. doi: 10.1016/j.asieco.2022.101570

[43] Fredrickson BL. The role of positive emotions in positive psychology: The broaden-and-build theory of positive emotions. American Psychologist. 2001;56: 218–226. 11315248 10.1037//0003-066x.56.3.218 PMC3122271

[44] Yan C, Wang J, Wang Z, Chan KC. Awe culture and corporate social responsibility: Evidence from China. Accounting & Finance. 2023. doi: 10.1111/acfi.13048

[45] Lei G, Wang W, Yu J, Chan KC. Cultural diversity and corporate tax avoidance: evidence from Chinese private enterprises. J Bus Ethics. 2022;176: 357–379.

[46] Bi K, Huang P, Wang X. Innovation performance and influencing factors of low-carbon technological innovation under the global value chain: A case of Chinese manufacturing industry. Technol Forecast Soc Change. 2016;111: 275–284.

[47] Zhou ZJ, Lei L. Business Environment Optimization and Corporate ESG Performance. Journal of Xiamen University(Arts & Social Sciences). 2022;48: 151–165. (In Chinese).

[48] Qiu B, Yu J, Chan KC. Does social trust restrain firm financing violations? Evidence from China. Accounting & Finance. 2021;61: 543–560. (In Chinese).

[49] Ferrell A, Liang H, Renneboog L. Socially responsible firms. Journal of Financial Economics. 2016;122: 585–606.

[50] Wang H, Shen H, Li S. ESG performance and stock price fragility. Financ Res Lett. 2023;56. doi: 10.1016/j.frl.2023.104101

[51] Wang S, Esperança JP. Can digital transformation improve market and ESG performance? Evidence from Chinese SMEs. J Clean Prod. 2023;419. doi: 10.1016/j.jclepro.2023.137980

[52] Sun N, Tao Y, Leng X, Li J. Social credit and corporate leverage: Evidence from a natural experiment in China. Manage Decis Econ. 2023;44: 3962–3978.

[53] Qiao F, Wen W. Research on the Impact of Social Credit System Reform on Corporate Green Innovation. Chinese Journal of Management. 2023;20: 1189–1197. (In Chinese).

[54] Dyck A, Lins KV, Roth L, Wagner HF. Do institutional investors drive corporate social responsibility? International evidence. Journal of Financial Economics. 2019;131: 693–714.

[55] Bai X, Han J, Ma Y, Zhang W. ESG performance, institutional investors’ preference and financing constraints: Empirical evidence from China. Borsa Istanbul Review. 2022;22: 157–168.

