# Correction: Can the improvement of the social credit environment enhance corporate ESG scores?

**DOI:** 10.1371/journal.pone.0309385

**Published:** 2024-08-20

**Authors:** Chao Han, Baoqi Chen

There is an error in affiliation for authors Chao Han and Baoqi Chen. The correct affiliation is: School of Economics, Shandong University of Finance and Economics, Jinan, China.

## References

[1] Han C, Chen B (2024) Can the improvement of the social credit environment enhance corporate ESG scores? PLOS ONE 19(3): e0300247. 10.1371/journal.pone.0300247 38530854 PMC10965064

